# Supplementary material for: Gait Characteristics and Cognitive Function in Middle-Aged Adults with and without Type 2 Diabetes Mellitus: Data from ENBIND
Source: Sensors (Basel). 2022 Jul 30;22(15):5710. doi: 10.3390/s22155710 (PMC9370923; doi:10.3390/s22155710)
Supplement: Supplementary file 1 [file sensors-22-05710-s001.zip › sensors-1790456-supplementary.pdf]

Supplementary Files

Supplementary Table S1. Pearson correlation coefficient (R^2) between gait variables and MoCA scores for the left foot. The correlation value varies between -1 and +1 with 0 implying no correlation. Correlating gait variables ($-0.3 \geq R^2 \geq 0.3$) with corresponding p value < 0.05 are marked bold.

MoCA variable	Visuospatial and executive	Naming	Attention	Language	Abstraction	Delayed Recall	Orientation	Total
Normal walk								
Velocity (m/s)	**0.35 (p<0.05)**	0.00 (p=0.97)	0.20 (p<0.05)	**0.35 (p<0.05)**	0.27 (p<0.05)	0.29 (p<0.05)	-0.10 (p=0.25)	**0.45 (p<0.05)**
Swing time (s)	0.01 (p=0.93)	0.08 (p=0.36)	0.01 (p=0.93)	0.08 (p=0.35)	0.02 (p=0.83)	-0.04 (p=0.65)	0.04 (p=0.62)	-0.03 (p=0.74)
Stance time (s)	0.06 (p=0.52)	0.11 (p=0.22)	-0.07 (p=0.46)	0.06 (p=0.53)	-0.03 (p=0.72)	-0.16 (p=0.07)	-0.05 (p=0.61)	-0.08 (p=0.34)
Stride time (s)	0.04 (p=0.63)	0.04 (p=0.64)	-0.04 (p=0.61)	-0.02 (p=0.83)	-0.01 (p=0.89)	-0.18 (p<0.05)	0.00 (p=0.96)	-0.1 (p=0.25)
Stride time variability (CoV)	-0.1 (p=0.27)	0.01 (p=0.90)	0.02 (p=0.79)	-0.11 (p=0.23)	0.05 (p=0.55)	-0.22 (p=0.01)	0.09 (p=0.29)	-0.16 (p=0.06)
Complexity index	-0.09 (p=0.32)	-0.01 (p=0.92)	-0.01 (p=0.88)	-0.18 (p<0.05)	0.12 (p=0.18)	-0.14 (p=0.11)	0.03 (p=0.7)	-0.13 (p=0.14)
Fast walk								
Velocity (m/s)	**0.34 (p<0.05)**	-0.05 (p=0.6)	0.20 (p<0.05)	0.23 (p<0.05)	0.11 (p=0.21)	**0.30 (p<0.05)**	-0.05 (p=0.55)	**0.41 (p<0.05)**
Swing time (s)	-0.04 (p=0.67)	-0.02 (p=0.82)	-0.03 (p=0.75)	-0.11 (p=0.20)	0.02 (p=0.80)	-0.12 (p=0.16)	0.08 (p=0.38)	-0.11 (p=0.23)
Stance time (s)	0.02 (p=0.86)	0.07 (p=0.41)	-0.07 (p=0.46)	-0.01 (p=0.95)	0.03 (p=0.71)	-0.19 (p<0.05)	-0.03 (p=0.73)	-0.12 (p=0.16)
Stride time (s)	0.00 (p=0.99)	0.03 (p=0.75)	-0.05 (p=0.57)	-0.09 (p=0.34)	0.03 (p=0.75)	-0.23 (p<0.05)	0.01 (p=0.88)	-0.17 (p=0.06)
Stride time variability (CoV)	0.1 (p=0.25)	0.03 (p=0.70)	0.08 (p=0.37)	0.03 (p=0.76)	0.06 (p=0.47)	-0.04 (p=0.63)	0.1 (p=0.24)	0.06 (p=0.50)
Complexity index	-0.09 (p=0.31)	0.04 (p=0.62)	0.03 (p=0.76)	-0.19 (p=0.03)	0.16 (p=0.08)	-0.16 (p=0.07)	-0.05 (p=0.56)	-0.14 (p=0.10)
Dual-task walk								
Velocity (m/s)	0.28 (p<0.05)	0.05 (p=0.59)	0.16 (p=0.07)	0.22 (p<0.05)	0.11 (p=0.23)	**0.35 (p<0.05)**	0.04 (p=0.66)	**0.42 (p<0.05)**
Swing time (s)	-0.09 (p=0.32)	-0.03 (p=0.77)	-0.02 (p=0.80)	-0.11 (p=0.23)	0.02 (p=0.85)	-0.14 (p=0.11)	0.11 (p=0.24)	-0.13 (p=0.14)
Stance time (s)	0.00 (p=0.98)	-0.04 (p=0.62)	-0.07 (p=0.46)	-0.02 (p=0.79)	0.01 (p=0.88)	-0.18 (p<0.05)	-0.01 (p=0.87)	-0.13 (p=0.13)
Stride time (s)	-0.04 (p=0.69)	-0.04 (p=0.62)	-0.07 (p=0.44)	-0.08 (p=0.36)	0.02 (p=0.85)	-0.2 (p<0.05)	0.04 (p=0.65)	-0.16 (p=0.07)
Stride time variability (CoV)	-0.05 (p=0.57)	-0.02 (p=0.80)	-0.06 (p=0.50)	-0.03 (p=0.77)	-0.08 (p=0.39)	-0.02 (p=0.82)	0.06 (p=0.51)	-0.06 (p=0.51)
Complexity index	-0.2 (p<0.05)	-0.01 (p=0.91)	-0.12 (p=0.19)	-0.09 (p=0.32)	0.01 (p=0.94)	-0.20 (p<0.05)	-0.02 (p=0.82)	-0.23 (p<0.01)

Supplementary Table S2. Pearson correlation coefficient (R^2) between gait variables and MoCA scores for the right foot. The correlation value varies between -1 and +1 with 0 implying no correlation. Correlating gait variables ($-0.3 \geq R^2 \geq 0.3$) with corresponding p value < 0.05 are marked bold.

MoCA variable	Visuospatial and executive	Naming	Attention	Language	Abstraction	Delayed Recall	Orientation	Total
Normal walk								
Velocity (m/s)	0.28 (p<0.05)	0.03 (p=0.77)	0.16 (p=0.08)	**0.31 (p<0.05)**	0.20 (p<0.05)	0.20 (p<0.05)	-0.1 (p=0.29)	**0.35 (p<0.05)**
Swing time (s)	0.13 (p=0.17)	-0.10 (p=0.29)	0.04 (p=0.63)	-0.15 (p=0.11)	0.04 (p=0.65)	-0.02 (p=0.84)	0.05 (p=0.57)	0.02 (p=0.85)
Stance time (s)	-0.03 (p=0.78)	0.13 (p=0.16)	-0.06 (p=0.53)	-0.06 (p=0.55)	-0.06 (p=0.48)	-0.1 (p=0.26)	-0.01 (p=0.87)	-0.1 (p=0.27)
Stride time (s)	0.07 (p=0.46)	0.042 (p=0.64)	-0.01 (p=0.89)	-0.06 (p=0.50)	-0.01 (p=0.92)	-0.09 (p=0.32)	0.01 (p=0.93)	-0.04 (p=0.64)
Stride time variability (CoV)	-0.04 (p=0.68)	-0.01 (p=0.94)	0.08 (p=0.37)	-0.05 (p=0.57)	0.09 (p=0.31)	-0.04 (p=0.66)	0.07 (p=0.47)	0 (p=0.96)
Complexity index	-0.14 (p=0.13)	0.03 (p=0.79)	-0.03 (p=0.76)	-0.24 (p<0.05)	0.01 (p=0.92)	-0.12 (p=0.20)	0.11 (p=0.21)	-0.17 (p=0.07)
Fast walk								
Velocity (m/s)	**0.37 (p<0.05)**	-0.07 (p=0.44)	0.25 (p=0.01)	0.28 (p<0.05)	0.13 (p=0.16)	0.28 (p<0.05)	-0.02 (p=0.82)	**0.43 (p<0.05)**
Swing time (s)	0.01 (p=0.95)	-0.14 (p=0.11)	0.00 (p=0.96)	-0.13 (p=0.15)	0.05 (p=0.62)	-0.05 (p=0.55)	0.02 (p=0.85)	-0.05 (p=0.55)
Stance time (s)	-0.07 (p=0.47)	0.11 (p=0.22)	-0.13 (p=0.16)	-0.12 (p=0.19)	-0.03 (p=0.76)	**-0.31 (p<0.05)**	0 (p=0.99)	-0.26 (p<0.05)
Stride time (s)	-0.05 (p=0.59)	0.02 (p=0.81)	-0.1 (p=0.3)	-0.15 (p=0.10)	0.00 (p=0.97)	-0.26 (p<0.05)	-0.01 (p=0.92)	-0.23 (p<0.05)
Stride time variability (CoV)	0.01 (p=0.95)	0.07 (p=0.47)	-0.02 (p=0.84)	-0.08 (p=0.37)	-0.05 (p=0.61)	-0.16 (p=0.09)	0.04 (p=0.64)	-0.11 (p=0.22)
Complexity index	-0.07 (0.48)	-0.01 (p=0.89)	-0.13 (p=0.15)	-0.27 (p<0.05)	0.12 (p=0.21)	-0.20 (p<0.05)	0.03 (p=0.71)	-0.21 (p<0.05)
Dual-task walk								
Velocity (m/s)	0.27 (p<0.05)	0.06 (p=0.51)	0.18 (p<0.05)	0.25 (p<0.05)	0.12 (p=0.17)	**0.35 (p<0.05)**	0.06 (p=0.48)	**0.43 (p<0.05)**
Swing time (s)	0.09 (p=0.31)	-0.1 (p=0.29)	-0.05 (p=0.55)	0.02 (p=0.84)	0.15 (p=0.11)	-0.12 (p=0.20)	0.06 (p=0.50)	-0.02 (p=0.85)
Stance time (s)	-0.13 (p=0.14)	0 (p=0.97)	-0.09 (p=0.31)	-0.08 (p=0.38)	-0.02 (p=0.86)	-0.26 (p<0.05)	0.01 (p=0.95)	-0.24 (p=0.01)
Stride time (s)	-0.07 (p=0.42)	-0.03 (p=0.73)	-0.09 (p=0.35)	-0.06 (p=0.49)	0.03 (p=0.71)	-0.25 (p<0.01)	0.02 (p=0.81)	-0.20 (p<0.05)
Stride time variability (CoV)	-0.09 (p=0.31)	0 (p=0.96)	-0.05 (p=0.62)	-0.02 (p=0.87)	-0.05 (p=0.59)	-0.14 (p=0.12)	0.04 (p=0.68)	-0.13 (p=0.15)
Complexity index	-0.1 (p=0.26)	-0.08 (p=0.38)	-0.11 (p=0.22)	-0.19 (p<0.05)	-0.02 (p=0.81)	-0.24 (p<0.05)	-0.02 (p=0.81)	-0.25 (p<0.01)

Supplementary Table S3. Pearson correlation coefficient (R^2) between gait variables and CANTAB scores for the left foot. The correlation value varies between -1 and +1 with 0 implying no correlation. Correlating gait variables ($-0.3 \geq R^2 \geq 0.3$) with corresponding p value < 0.05 are marked bold.

CANTAB	Paired Associates Learning	Spatial Working Memory	Pattern Recognition Memory	Reaction Time	One Touch Stokings of Cambridge	Rapid Visual Processing
Normal walk						
Velocity (m/s)	0.24 (p<0.05)	-0.16 (p=0.09)	0.22 (p<0.05)	-0.12 (p=0.19)	0.04 (p=0.68)	0.17 (p=0.07)
Swing time (s)	-0.06 (p=0.55)	0.00 (p=0.97)	0.07 (p=0.44)	0.11 (p=0.25)	-0.11 (p=0.25)	-0.07 (p=0.48)
Stance time (s)	0.12 (p=0.18)	0.11 (p=0.24)	-0.01 (p=0.87)	0.21 (p<0.05)	-0.06 (p=0.55)	-0.13 (p=0.16)
Stride time (s)	0.09 (p=0.34)	0.11 (p=0.25)	0.02 (p=0.83)	0.24 (p<0.05)	-0.08 (p=0.36)	-0.16 (p=0.09)
Stride time variability (CoV)	0.02 (p=0.83)	0.08 (p=0.36)	-0.12 (p=0.18)	0.04 (p=0.66)	0.05 (p=0.57)	-0.16 (p=0.08)
Complexity index	-0.07 (p=0.43)	0.00 (p=0.98)	-0.14 (p=0.12)	-0.08 (p=0.38)	-0.06 (p=0.53)	-0.07 (p=0.46)
Fast walk						
Velocity (m/s)	0.15 (p=0.11)	-0.19 (p<0.05)	0.24 (p<0.01)	-0.18 (p<0.05)	0.01 (p=0.91)	0.11 (p=0.23)
Swing time (s)	-0.07 (p=0.44)	-0.07 (p=0.42)	0.01 (p=0.89)	0.07 (p=0.43)	-0.07 (p=0.45)	0.04 (p=0.63)
Stance time (s)	0.1 (p=0.29)	0.12 (p=0.19)	0.00 (p=0.96)	0.18 (p<0.05)	-0.11 (p=0.24)	-0.12 (p=0.19)
Stride time (s)	0.02 (p=0.86)	0.07 (p=0.46)	0.01 (p=0.94)	0.19 (p<0.05)	-0.12 (p=0.19)	-0.06 (p=0.53)
Stride time variability (CoV)	0.13 (p=0.16)	-0.14 (p=0.14)	0.06 (p=0.48)	0.07 (p=0.47)	-0.21 (p<0.05)	-0.08 (p=0.36)
Complexity index	-0.17 (p=0.06)	0.07 (p=0.47)	-0.22 (p=0.02)	0.02 (p=0.85)	-0.07 (p=0.47)	-0.07 (p=0.43)
Dual-task walk						
Velocity (m/s)	0.00 (p=0.97)	0.05 (p=0.56)	0.27 (p<0.05)	-0.05 (p=0.56)	0.05 (p=0.60)	0.04 (p=0.65)
Swing time (s)	-0.01 (p=0.99)	-0.18 (p<0.05)	0.01 (p=0.88)	0.06 (p=0.48)	-0.03 (p=0.73)	-0.01 (p=0.95)
Stance time (s)	0.17 (p=0.07)	-0.18 (p=0.06)	-0.06 (p=0.50)	0.13 (p=0.15)	0.0 (p=0.92)	0.02 (p=0.86)
Stride time (s)	0.13 (p=0.16)	-0.23 (p<0.05)	-0.03 (p=0.76)	0.14 (p=0.12)	0.01 (p=0.93)	0.05 (p=0.62)
Stride time variability (CoV)	0.09 (p=0.35)	-0.29 (p<0.05)	-0.03 (p=0.75)	-0.02 (p=0.86)	-0.11 (p=0.26)	0.12 (p=0.21)
Complexity index	-0.07 (p=0.47)	-0.01 (p=0.91)	-0.27 (p<0.05)	0.09 (p=0.34)	-0.07 (p=0.43)	-0.03 (p=0.74)

Supplementary Table S4. Pearson correlation coefficient (R^2) between gait variables and CANTAB scores for the right foot. The correlation value varies between -1 and +1 with 0 implying no correlation. Correlating gait variables ($-0.3 \geq R^2 \geq 0.3$) with corresponding p value < 0.05 are marked bold.

CANTAB	Paired Associates Learning	Spatial Working Memory	Pattern Recognition Memory	Reaction Time	One Touch Stokings of Cambridge	Rapid Visual Processing
Normal walk						
Velocity (m/s)	0.16 (p=0.09)	-0.12 (p=0.23)	0.28 (p<0.05)	-0.09 (p=0.35)	0.04 (p=0.65)	0.16 (p=0.09)
Swing time (s)	-0.04 (p=0.66)	-0.05 (p=0.64)	-0.06 (p=0.52)	-0.01 (p=0.92)	-0.13 (p=0.19)	-0.05 (p=0.61)
Stance time (s)	0.1 (p=0.30)	0.01 (p=0.96)	0.01 (p=0.92)	0.11 (p=0.25)	-0.04 (p=0.66)	-0.18 (p=0.06)
Stride time (s)	0.11 (p=0.24)	-0.03 (p=0.73)	0.01 (p=0.95)	0.1 (p=0.29)	-0.1 (p=0.28)	-0.16 (p=0.09)
Stride time variability (CoV)	-0.03 (p=0.75)	0.07 (p=0.49)	0.04 (p=0.65)	-0.09 (p=0.35)	0.09 (p=0.34)	-0.19 (p<0.05)
Complexity index	-0.1 (p=0.28)	0.06 (p=0.53)	-0.16 (p=0.09)	0.00 (p=0.97)	-0.03 (p=0.79)	-0.03 (p=0.76)
Fast walk						
Velocity (m/s)	0.21 (p<0.05)	-0.22 (p<0.05)	0.23 (p<0.05)	-0.16 (p=0.10)	-0.03 (p=0.73)	0.06 (p=0.55)
Swing time (s)	-0.13 (p=0.18)	-0.03 (p=0.79)	0.04 (p=0.67)	0.09 (p=0.37)	-0.06 (p=0.56)	0.00 (p=0.98)
Stance time (s)	0.03 (p=0.71)	0.06 (p=0.54)	-0.03 (p=0.73)	0.17 (p=0.07)	-0.01 (p=0.93)	-0.01 (p=0.89)
Stride time (s)	-0.02 (p=0.80)	0.02 (p=0.80)	-0.01 (p=0.94)	0.16 (p=0.09)	-0.03 (p=0.74)	0.00 (p=0.98)
Stride time variability (CoV)	-0.02 (p=0.88)	-0.02 (p=0.82)	-0.09 (p=0.33)	0.08 (p=0.40)	-0.03 (p=0.75)	-0.15 (p=0.12)
Complexity index	-0.21 (p<0.05)	-0.04 (p=0.67)	-0.26 (p<0.05)	0.04 (p=0.68)	0.07 (p=0.48)	0.04 (p=0.68)
Dual-task walk						
Velocity (m/s)	0.09 (p=0.36)	0.03 (p=0.76)	0.2 (p<0.05)	-0.16 (p=0.09)	0.13 (p=0.18)	0.09 (p=0.35)
Swing time (s)	0.13 (p=0.16)	-0.27 (p<0.05)	0.03 (p=0.78)	0.1 (p=0.30)	0.03 (p=0.75)	0.01 (p=0.94)
Stance time (s)	0.07 (p=0.49)	-0.20 (p<0.05)	-0.07 (p=0.47)	0.14 (p=0.15)	0.02 (p=0.83)	-0.03 (p=0.79)
Stride time (s)	0.09 (p=0.37)	-0.24 (p<0.05)	-0.06 (p=0.54)	0.14 (p=0.13)	0.01 (p=0.89)	-0.02 (p=0.80)
Stride time variability (CoV)	0.03 (p=0.75)	-0.23 (p<0.05)	-0.08 (p=0.42)	0.05 (p=0.61)	0.00 (p=1.00)	0.00 (p=0.98)
Complexity index	-0.05 (p=0.58)	-0.07 (p=0.46)	-0.26 (p<0.05)	0.08 (p=0.38)	-0.01 (p=0.88)	-0.04 (p=0.66)
